# Same-Day Discharge After Percutaneous Coronary Intervention in Patients With Acute Coronary Syndromes: A Pilot Study

**DOI:** 10.1016/j.jscai.2025.103728

**Published:** 2025-06-24

**Authors:** Attilio Galhardo, Siddhartha Mengi, Vitor S. Netto, Renato D. Lopes, Adriano Caixeta

**Affiliations:** a Division of Cardiology, Escola Paulista de Medicina, Universidade Federal de São Paulo, São Paulo, Brazil; b Division of Cardiology, Université Laval, Quebec, Canada; c Division of Cardiology, Duke Clinical Research Institute, Duke University, Durham, North Carolina; d Department of Cardiology, Hospital Israelita Albert Einstein, São Paulo, Brazil

**Keywords:** acute coronary syndrome, percutaneous coronary intervention, same-day discharge

Since the early 2000s, the strategy of same-day discharge (SDD) after elective percutaneous coronary intervention (PCI) has become a well-established practice. This shift—primarily driven by advancements in newer-generation drug-eluting stents, enhanced pharmacotherapy, operator experience, and improved access-site practices—has significantly increased the safety of PCI.^1^ The feasibility and safety of SDD following PCI in patients with stable angina have been examined in several studies, including both randomized controlled trials and observational studies. As a result, numerous cardiac catheterization laboratories around the world have implemented outpatient protocols to facilitate SDD for eligible patients.^1^, ^2^, ^3^ Large PCI registries from several developed countries have shown a steady increase in SDD PCI procedures^2^; however, the SDD strategy in patients with acute coronary syndrome (ACS) undergoing PCI remains less explored.^4^^,^^5^

In developing countries, where primary PCI facilities are scarce, fibrinolytic and/or pharmacoinvasive strategies remain prevalent for managing ST-segment elevation myocardial infarction, often resulting in extended hospital stays while patients await invasive procedures at tertiary centers.^5^ Furthermore, initial hospitalization in hospitals lacking PCI capabilities often leads to additional delays, as ACS patients must await transfer to tertiary care facilities for definitive treatment. During this initial waiting period, most patients are medically stabilized, potentially making them candidates for SDD following uncomplicated PCI.^5^

The present pilot study aimed to evaluate the feasibility of SDD within 6 hours postprocedure in low-risk, stabilized ACS patients undergoing uncomplicated, noncomplex PCI. From November 2020 to August 2021, 20 consecutive ACS patients, all stable at the time of PCI (≥72 hours postindex event), were included. Inclusion required a complication-free PCI and no clinically significant postprocedural events, such as major access-site bleeding, hematoma, new or worsening electrocardiographic abnormalities, recurrent angina, hemodynamic instability, or stent thrombosis. Patients were also required to have sufficient social support and logistical capacity for timely hospital access in the event of an emergency. Patients were excluded if they had severe left ventricular systolic dysfunction (left ventricular ejection fraction <40%), advanced heart failure (NYHA class III or IV), prior PCI within the preceding month, a residual nonculprit lesion requiring treatment within 7 days of the index PCI, or complex PCI—including left main interventions or bifurcation PCI requiring a 2-stent technique. Additional exclusion criteria included intracoronary thrombus, side branch loss, major dissection, saphenous vein graft PCI, and PCI involving calcium modification. To ensure patient safety in this pilot study, we implemented a comprehensive predischarge education program that included detailed counseling on medication regimens, lifestyle modifications, and clear instructions regarding follow-up care at 24 hours and 7 days. For comparison, we selected 80 patients from our data set who underwent PCI for ACS during the same period. First, we applied the same inclusion and exclusion criteria used for the SDD group. Second, we performed a 1:4 propensity score matching analysis using variables including age, sex, presence of diabetes mellitus, systemic arterial hypertension, dyslipidemia, number of stents implanted, clinical presentation, and culprit artery. A mean distance of 0.127 with an SD of 0.088 was obtained, indicating low variability between matched pairs. Subclass distribution was balanced, with the largest subclasses containing 5 individuals each, reflecting careful matching across participant characteristics. For continuous variables, the *t* test was used to compare means; for categorical variables, the χ^2^ test was applied to assess independence between categories.

Table 1 presents the demographic and angiographic characteristics of the 2 groups. At the 7-day follow-up, there were no major adverse cardiovascular events (a composite of death, target vessel myocardial infarction, or ischemia-driven target lesion revascularization) or BARC 3 to 5 bleeding in both groups. The median hospitalization duration after PCI was 7.45 ± 1.05 hours in the SDD group, compared with 54.30 ± 164.06 hours in the overnight group (*P* = .01).

**Table 1:** Demographic and angiographic characteristics.

	SDD (n = 20)	Control (n = 80)	*P* value
Age, y	58.90 ± 8.42	59.35 ± 9.83	.84
Female sex	6 (30)	22 (27.50)	>.99
Hypertension	11 (55)	49 (61.25)	.80
Diabetes	6 (30)	26 (32.50)	>.99
Dyslipidemia	6 (30.00)	27 (33.75)	.96
LVEF, %	53.6 ± 4.5	49.5 ± 5.0	<.01
Previous MI	1 (5)	19 (23.7)	.03
Acute coronary syndromes			0.56
STEMI^a^	5 (25)	28 (35)	
Non-STEMI	15 (75)	52 (65)	
Culprit artery			.99
LAD	9 (45)	37 (46.25)	
RCA	8 (40)	32 (40)	
Circumflex	3 (15)	11 (13.7)	
Time from admission to PCI, d	9.2 ± 2.2	9.7 ± 2.5	.4
Length of stay after PCI, h	7.45 ± 1.05	54.30 ± 164.06	.01
Total length of stay, d	9.51 ± 2.2	11.96 ± 7.3	.10
Radial access	18 (90)	20 (75)	.15
No. of stents	1.65 ± 0.67	1.61 ± 0.65	.82
Medications			
Aspirin	20 (100)	80 (100)	–
Clopidogrel	20 (100)	80 (100)	–

Values are mean ± SD or n (%).

ACS, acute coronary syndromes; LAD, left anterior descending; LVEF, left ventricular ejection fraction; MI, myocardial infarction; non-STEMI, non-ST-segment elevation myocardial infarction; PCI, percutaneous coronary intervention; RCA, right coronary artery; SDD, same-day discharge; STEMI, ST-segment elevation myocardial infarction.

a All STEMI patients in both groups underwent fibrinolysis.

This study highlights a markedly different geographic and logistical context compared to prior studies conducted in high-income countries.^4^^,^^5^ By situating our findings within this underrepresented health care setting, we believe our study contributes meaningful real-world data that may inform clinical practice and health policy in similarly resource-constrained environments.

The main limitations of this study are its nonrandomized design, small sample size, very short-term (7-day) follow-up without 30-day outcome data, and limited generalizability, as it applies only to a selected population of low-risk ACS patients.

In conclusion, this exploratory study holds particular relevance in settings where early invasive treatment with primary PCI is not widely available. The findings suggest that SDD may be a feasible and safe strategy for stabilized, selected, low-risk ACS patients, offering a viable alternative in resource-constrained environments. Larger, more comprehensive studies are warranted to further validate these results.
